# Toxic Metals (As, Cd, Ni, Pb) Impact in the Most Common Medicinal Plant (*Mentha piperita*)

**DOI:** 10.3390/ijerph18083904

**Published:** 2021-04-08

**Authors:** Cristina Dinu, Stefania Gheorghe, Anda Gabriela Tenea, Catalina Stoica, Gabriela-Geanina Vasile, Roxana Luisa Popescu, Ecaterina Anca Serban, Luoana Florentina Pascu

**Affiliations:** 1 National Research and Development Institute for Industrial Ecology ECOIND Bucharest, 71-73 Drumul Podul Dambovitei Street, 060652 Bucharest, Romania; cristina.dinu@incdecoind.ro (C.D.); anda.tenea@incdecoind.ro (A.G.T.); catalina.stoica@incdecoind.ro (C.S.); gabriela.vasile@incdecoind.ro (G.-G.V.); anca.serban@incdecoind.ro (E.A.S.); ecoind@incdecoind.ro (L.F.P.); 2 Science Faculty, Chemistry Department, University of Craiova, 107i Bucharest Street, 200478 Craiova, Romania; 3 National Research and Development Institute for Industrial Ecology ECOIND Ramnicu Valcea Branch, 182 Stirbei Voda Street, 240588 Ramnicu Valcea, Romania; roxana.popescu@incdecoind.ro

**Keywords:** *Mentha piperita*, metals, translocation

## Abstract

This study aimed to evaluate the behavior of *Mentha piperita* under Cd, Pb, Ni, and As soil contamination and their transfer from soil in plants as well as translocation in the roots/stems/leaves system compared with a control without metal addition. The mint seedlings were exposed for a three-month period using two metal mixtures in the same concentrations such as AsCd and AsCdNiPb (23.7 mg/kg As, 5 mg/kg Cd, 136 mg/kg Ni, and 95 mg/kg Pb). The results of metal concentration in plants showed that Cd, Ni, and Pb were accumulated in different parts of the plant, except for As. In plants organs, the order of metal accumulation was roots > stems > leaves. No significant impact on the growth, development, and chlorophyll content compared to the control was observed in the first month of exposure. After three months of exposure, phytotoxic effects occurred. Generally, the transfer coefficients and translocation factors values were less than 1, indicating that *Mentha piperita* immobilized the metals in root. The laboratory experiments highlighted that for a short period of time, *Mentha piperita* has the capacity to stabilize the metals at the root level and was a metal-tolerant plant when using a garden rich-substrate.

## 1. Introduction 

The presence of metals in the environment represents a concern for the safety of the food and implicitly for human life. In natural systems, heavy metals come from rocks, ores, volcanoes, and from the release of metals during the weather that leads to soil formation [1]. A very acute problem occurs when the heavy metals concentration increases significantly in certain ecosystems due to anthropogenic activities [2,3,4,5,6,7].

Heavy metals interact strongly with the soil matrix and may become mobile as a result of changing environmental conditions [8]. Considering the fact that the soil represents a living system consisting in various organisms including plants, an increase of contaminants can adversely affect the normal growth or development of plants and finally the safety of plant products. The plant are soil-dependent photosynthetic organisms, which have the ability to accumulate trace elements, especially heavy metals, due to their ability to tolerate potentially toxic ions in the environment [9]. Human and animal diets, as well as health care programs, are based on various vegetable foods and medicinal herbs, leading to the bio magnification process of some accumulative contaminants in the final trophic chain. From the ancient times and likewise nowadays, the medicinal plants are the primary source for prevention, improving of symptoms, and the treatment in various diseases. According to the World Health Organization (WHO), approximately 80% of the population living in the outlying areas use only herbs to treat multiple diseases [10,11]. When using herbs in the treatment of certain conditions, it should be noted that in addition to the pharmacological effect, they can be toxic if they contain toxic contaminants such as pesticides or heavy metals. It is considered that prolonged treatment with plants with a heavy metal content such as: lead (Pb), cadmium (Cd), zinc (Zn), and nickel (Ni) above the maximum allowed limit can cause severe health problems, including dermatitis, poisoning, organ dysfunctions, cancer, mental retardation, damage to nervous system, anemia, etc. [12]. In addition, plants can be easily contaminated with heavy metals during both cultivation and processing [13].

According to the WHO, the maximum allowed limits sets in medicinal herbs are 0.3 mg/kg dry weight (d.w.) for Cd, 1 mg/kg d.w for arsenic (As), and 10 mg/kg d.w. for Pb [14,15]. In addition, WHO documents specified permissible limits for As such as 5 mg/kg d.w. in Canada, 2 mg/kg d.w. in China, and 4 mg/kg d.w. in Thailand [16]. The same organization recommended a maximum limit of 10 μg/L of As in the drinking water, which is a very small concentration that can induce toxicity [17]. Moreover, European Commission Regulation no. 1881/2006 for foodstuff contamination set for Pb 0.1 mg/kg d.w. and Cd 0.2 mg/kg d.w. in fresh herbs [18].

Depending on their role in the organism body, heavy metals can be essential and non-essential. Essential metals or micronutrients, such as Cr, Co, Cu, Mn, Mo, Ni, Fe, Se, and Zn are required for optimal functioning of biological and biochemical processes, including humans. Non-essential metals such as As, Cd, Hg, and Pb have no known biological function. Heavy metals are not biodegradable, which is why they can be toxic even at low concentrations, representing a serious threat to both the environment and human health [19].

Lead is a very common contaminant in industrial wastewater being used in many industrial applications such as the manufacturing of batteries, pigment for paint, photographic materials, auto industry, etc. [20,21]. In agriculture, Pb limits crop productivity [22] and could increase the production of reactive oxygen species inducing the oxidative stress in plants [23]. Lead was detected in different market products (pharmacy, herbal store, traditional market, supermarket) ranging from 0.03 to 1.11 mg/kg, but also Cd (0.18 to 0.28 mg/kg) and Ni (1.01 to 1.96 mg/kg) according to Rubio et al. [24].

Cadmium is generally derived from human activities (mining, metallurgy, waste combustion, and abusive pesticides and fertilizer use). It is very toxic for aquatic and terrestrial organisms, which has been well studied [25,26]. Increased concentrations of Cd in plants can lead to reduced photosynthesis, decreased water absorption, and lower nutrient uptake. Plants grown in soils containing high amounts of Cd are affected by growth inhibition, root drying, and then plant death [25,27].

Another toxic metal is arsenic derived by industrial activities or from the use of fertilizers. As occurs most commonly in the environment in trivalent and pentavalent oxidation states, which are found in both organic and inorganic compounds. Arsenate (AsO_4_^3−^) and arsenite (AsO_3_^3−^) are the most soluble inorganic forms found in water and soil [19,28]. The impact on the environment is associated with the high mobility of this element [29]. The concentrations of different forms of arsenic in soil, as well as its mobility in the water–soil–plant system are affected by the physical, chemical, and biochemical properties of the soil such as redox potential, pH, texture, presence of changeable ions, biological activity, and organic matter content [30,31].

Nickel is an essential element for the growth and development of plants mainly for the activation of enzymes such as urea and glyoxalase. Nickel is also important in the germination process of seeds, in photosynthesis and in the metabolism of nitrogen [32], but it can become toxic for plants at high concentrations (more than 10 to 1000 mg/kg d.w. depend on plant sensitivity) [33].

In Romania, different sites contaminated with toxic metals were reported, mainly as a result of anthropogenic sources. Different types of soils and sediments collected from mining sites (Baia Mare (As, Cd) and Certej (As, Cd, Ni, Pb)), or from areas adjacent to metallurgical activities, such as Copsa Mica (Cd, Cu, Pb, Zn) or Zlatna (As, Cu, Pb, Zn) were highly contaminated [6,34,35,36].

In mining areas, where surface mining is either in operation or closed, the amounts of toxic metals discharged into the environment can be dangerously elevated. Thus, by leaching the contaminated areas with rainwater, both surface waters and sediments can be polluted. As a result, aquatic and riparian flora could be affected. Dinu et al. reported concentrations of metals ranging between 5.5 and 121 mg As/kg; 1.2 and 11.4 mg Cd/kg; 26 and 610 mg Ni/kg; 50 and 888 mg Pb/kg in soil samples collected from an area situated in Romania [37].

Since they have the capacity to concentrate in soil, heavy metals are easily transferred along the trophic chain. There are various studies on heavy metals accumulation in plants cultivated in contaminated soil, especially using plants with economic interest such as crop plants, food fresh plants, medicinal, or aromatic plants [15,26,38,39].

The phyto-pharmaceutical products based on *Geranium sps.*, *Mentha sps*, *Ocinum sps.*, *Matricaria sps.*, *Salvia sps.*, *Lavandula sps.*, and *Rosmarinus sps*. are widespread, and the plants are based from organic crops [39,40,41]. Janvi Pandey reported that certain medicinal plants are very good Cd, Pb, Zn, and Cr accumulators [39]. Since *Mentha sp*. medicinal or aromatic products are widely spread, the *Mentha* plants growing in metal contaminated site could affect the human health safety.

Various metals concentration such as Pb 0.65 ± 0.71 mg/kg (Spain), 1.83 to 2.58 mg/kg (Bulgaria), 2.41 mg/kg (Poland); Cd 0.22 ± 0.13 mg/kg (Spain), 0.09 mg/kg (Poland), 0.369 mg/kg (India), 0.022 to 0.12 mg/kg (Bulgaria), 0.02 mg/kg (Turkey); and Ni 4.84 ± 1.13 mg/kg (Spain), 2.99 mg/kg (Poland), 0.002 mg/kg (India), 3.52 mg/kg (Turkey) were detected in *Mentha piperita* herbal tea as reported by Rubio et al. [24].

*Mentha piperita* is part of the *Lamiaceae* family along with other aromatic plants used in medicinal products, foods, drinks, teas, cosmetically products, etc. Several species of mint have been described, but not all of them have therapeutic properties. The mint species, which are recognized to have medicinal properties, are *Mentha aquatica* (water mint), *Mentha viridis* or *Mentha spicata* (sweet mint), and a hybrid of the two species: *Mentha piperita*. All species of mint develop very well in cool places, partially covered by the sun. The growth of the plant is very fast. The plant has a roots system from which appear stems up to 40–130 cm high. The plant is multiplied by cuttings or stolons. The stems are red-purple and very branched, and the leaves are elongated-oval in shape, light green in color, and have serrated edges. In Europe, peppermint came into medicinal use for nausea, vomiting, and gastrointestinal disorders in the mid-eighteenth century. Extracts from *Mentha piperita* leaves have therapeutic efficiency in arsenic toxicity amelioration in Swiss Albino mice. It seems that this plant decreases the lipid peroxidation, scavenging free radicals, reducing genotoxicity, and exhibiting the hepatic effects caused by the arsenic contamination [28]. In addition to medicinal properties, mint is also known for insecticidal properties against mosquitoes, wasps, and beetles [42,43].

Moreover, in the last five years, more than 120 applicative experiments on *Mentha* species were performed in research areas such as plant science, food science, pharmacology, and biotechnology, in environmental sciences and ecology (https://webofknowledge.com, accessed on 30 July 2020).

This study aimed to evaluate the effect of toxic metals Cd, Ni, Pb, and As on *Mentha piperita* grown in an enriched soil in laboratory conditions by simulating a soil polluted by mining activities. The experiment was carried out over a period of three months, in order to assess the ability of *Mentha piperita* to accumulate the metals from the soil and to transfer them from the roots to the aerial parts (stem, leaves).

## 2. Materials and Methods

### 2.1. Plants and Soil Characteristics

Ten uniform mint (*Mentha piperita*) seedlings (from local agriculture) with initial size of about 20 ± 5 cm were placed in 5000 g soil for each control and polluted soil in different flower pots under laboratory conditions.

The soil control was a universal substrate enriched with nutritive elements provided by local producers, consisting of peat and humus for garden plants cultivation. An initial physical and chemical characterization of the control soil before the planting procedure was done.

The soil enrichment solutions were prepared using standard solutions of Cd, Ni, Pb, and As in 5% HNO_3_ dissolved in ultrapure water obtained using an Elix Technology Inside with a Quantum ICP Polishing Cartridge (Milli-Q, Molsteim, France) system, with a resistivity of at least 18 MΩ·cm (equivalent to a conductivity of less than 5.6 × 10^−5^ mS cm^−1^).

The soil treatment with metal solutions was performed at the beginning of the experiment (in May 2019), by watering, one week after seeding of the plants.

### 2.2. Experiment Characteristics

The laboratory soil contamination simulates the characteristics of industrial polluted soil by mining activities.

To reach the intervention threshold values for sensitive soils according to the Romanian legislation MAPPM (1997) for analyzed elements (As, Cd, Ni, Pb), the soils were spiked with different solutions. Three different experiments were noted as *M* (the control soil with no metals addition); *AsCd* (polluted soil with 23.7 mg/kg As and 5 mg/kg Cd); and *AsCdNiPb* (polluted soil with 23.7 mg/kg As, 5 mg/kg Cd, 136 mg/kg Ni and 95 mg/kg Pb).

The concentrations and mixture of toxic metals were established considering the real contamination circumstances prevailing in Romania to estimate the overall effects of metals on the plant development.

The enrichments were added taking into account the initial concentration of elements detected in the used substrate (Table 1). As, Cd, Ni, and Pb values in the control experiment (M) indicate normal values for soil quality [44].

The batch of experiments was performed in a laboratory using an acclimatization system (23 ± 2 °C) under natural light (12 h per day and night, day light intensity was 421 to 2411 lux) and relative humidity of about 23%. The pots were rotated and were mixed with a weekly frequency. To prevent the leakage of nutrients and metals from the substrate, plastic trays were used under each pot. The exposure period was about three months (May, June, and July). The plants were soaked with free chlorine tap water. An initial characterization of water before use was done. The watering was performed about three times per week in order to ensure a constant soil humidity for about 60% of the maximum moisture holding capacity of used soil.

### 2.3. Sampling of Soil and Chemical Analyses 

The metals (Cd, Ni, Pb, As) content was monthly detected both from soil (control soil/polluted soil) and plants (control/exposed plants), respectively. The soil samples for metal content detection were collected from two depths as follows: 0–6 cm and 6–12 cm. The depths were in accordance with the elongation of the peppermint plant roots. Up to 12 cm, the root maturation zone starts to develop; around this depth, the primary roots acquire lateral or hair roots.

The samples were air dried, homogenized, and sieved for analysis of the fraction less than 150 μm according to ISO 11464:2006 [45]. A Retsch RM 100 Mill (Haan, Germany), a Fritsch Analysette 3 Spartan vibratory sieve shaker (Idar, Oberstein), and a Kern ABT 220-50M analytical balance (Balingen, Germany) were used for the soil samples preparation.

Two grams of soil were digested with a mixture of nitric acid and hydrochloric acid (1:3 *v*/*v*) and then mineralized in an open system (Falc ceramic-glass heater, Treviglio, Italy) until the organic matter was mineralized. The mixture was filtered and washed with analytical pure water according to ISO 11466 [46] and to Vasile et al. [47]. The samples were analyzed for the metal content by inductive coupled plasma optical emission spectrometry (ICP-EOS, Avio 500 Perkin Elmer Spectrometer, Waltham, MA, USA).

The following chemical parameters were additionally analyzed using standardized methods: pH—SR EN ISO 10523:2012; conductivity—SR ISO 11265 + A1:1998, potassium (K)—SR EN 16170:2017, total nitrogen (N_total_)—SR ISO 11261:2000; total phosphorus (P_total_)—STAS 7184/14-79; total organic carbon (TOC)—SR EN 15936:2013, humus and total carbon (C_total_) SR ISO 10694:1998; organic chlorinate pesticides (SR ISO 10382:2007) and triazine pesticides (ISO 11264:2005(E)).

The results validity of nutrients, metals, and pH determinations in soil were verified using a sewage sludge Certified reference material VHG-SL1 (LGC Standards, Manchester, NH, USA).

For the results validity of total carbon, the followed Certified reference materials were used: Soil Standard Silty OAS (Elemental Microanalysis Ltd., Okehampton, UK) and harbor sediments. AGLAE 19M 9.2 (AGLAE Association, Giberville, France). For TOC, we used Soil Standard Clay B2184 (Elemental Microanalysis Ltd., Okehampton, UK).

### 2.4. Sampling of Plants and Metal Analyses

The harvested mint plants used for metal detection were washed firstly with tap water and secondly with distilled water. The plants were separated on roots, leaves, and stems and dried using a Lyophilizer Christ Alpha 1-2 LD (Martin Christ GmbH, Osterode, Germany) at −55 °C. After the drying procedure, the mint samples were thinly milled. About 0.2 to 0.5 g was weighed for metal content analysis. The mint samples were processed with 10 mL of HNO_3_ and 3 mL of H_2_O_2_ for 24 h at room temperature in order to mineralize organic matter (cold mineralization). The final mineralization at 180 °C (1800 W) for 15 min was performed using a Milestone Ethos Up microwave digester (Sorisole, Italy). The extract was filled up to a 25 mL volumetric flask, and the metal concentration was detected.

The content of metals in plant extracts was controlled using a simultaneous determination method with an ICP-EOS technique. The performance parameters of the applied method (detection limit, precision, and uncertainty) are presented in Table 2.

To assure the quality and validity of results, a certified reference material consisting of trace elements (As, Cd, Cr, Cu, Ni, Pb, and Zn) in lichen powder (BCR-482, Institute for Reference Materials and Measurements, Geel, Belgium) was used. The recovery percentages for all analyzed metals varied between 95.8 and 103.7% (Table 2).

### 2.5. Biometrical Measurements and Chlorophyll Detection

The biometrical measurements and chlorophyll detection were done only after one month of *Mentha piperita* plants metals exposure. The stem elongation, the number of leaves, and their length, width, and wet weight were analyzed. Due to the phytotoxic effects observed at the final of the test, only stems were measured.

The chlorophyll pigments (chlorophyll *a* and chlorophyll *b*) of mint samples were analyzed considering the methods described in Lichtenthaler and Buschmann [48], and Krishnan et al. [49]. The chlorophyll from the leaves samples was extracted in 80% acetone at 1:2.5 [*mass (g)*/*volume(mL)]* ratio. The leaves were cut into 1–2 cm pieces and homogenized. The leaves tissues were incubated in 80% buffered acetone on a mixing tube revolver Rotator D6050 (neoLabLine, Heidelgerg, Germany) for 12 h at 4 °C.

The extract was centrifuged for 3 min at 2545× *g* (centrifuge 5702R, Eppendorf AG Hamburg, Germany), and subsequently, the absorbance at 662 nm and 645 nm was measured using UV-VIS spectrometer Specord 205BU (Analytik Jena, Jena, Germany). Chlorophyll concentrations were calculated using the following equations:

(1) $$\mathrm{Chlorophyll}\;\mathrm{concentration}\;a\;(\mathrm{mg}/\mathrm{mL})\;=\left(12.25\times A_{662\;nm}\right)-(2.79\times A_{645\;nm})$$

(2) $$\mathrm{Chlorophyll}\;\mathrm{concentration}\;b\;(\mathrm{mg}/\mathrm{mL})\;=\left(21.5\times A_{645\;nm}\right)-(5.1\times A_{662\;nm}).$$

The sum of the two types of chlorophyll pigments represented the total chlorophyll concentration (mg/mL).

### 2.6. Data Analyses 

The results represented the average of the two separate experiments. The samples collected from each experiment were analyzed for metal detection as follows: in three replicates for soil and two replicates for plants. In addition, two replicates were performed for chlorophyll detection. The quantities of plants samples obtained after the drying process were in some cases not sufficient for three replicates.

The results were correlated and compared with control samples values and with the reference values for the soil and plant quality [14,16,44].

To evaluate the plants’ ability to accumulate metals from soil and to transfer them from the root to the aerial parts (stem, leaves), the transfer coefficient (TC) or bioaccumulation factor (BCF) and the translocation factor (TF) were calculated. The TC factor was defined as the ratio between the metal concentration in the roots and its content in the soil (results express in mg/kg) [50]. Olowoyo et al. stated that a TC value higher than 1 suggests metals accumulation, a TC value around 1 shows that the plant was not influenced by the metal, and a TC less than 1 indicates no metal uptake [51].

The TF factor was calculated as the ratio between the metal concentration detected in the upper aerial part and the metal concentration detected in the roots [50]. In this case, the values higher than 1 indicate that the plant effectively translocate metals from the root to the aboveground plant part.

These parameters were calculated taking into consideration the tested metals content (Cd, Ni, Pb) in control and polluted samples collected in May, June, and July, except for As, which was not detected in plants.

### 2.7. Statistical Analysis

The average and standard deviation (SD) values were calculated for all metal concentrations in soil (*n* = 6) and plant (*n* = 4), biometrical indicators (*n* = 12), and chlorophyll (*n* = 4). The Pearson correlation (*r*) was used for the correlation between experiments.

The interpretation of the obtained experimental data was performed using ANOVA ONE-WAY, depending on the value of *p* obtained. The value of *p* was considered insignificant for *p* > 0.05 (ns), significant for *p* < 0.05 (*), and very significant for *p* < 0.01 (**). Thus, the experimental data obtained for the soil depth (0–6 cm and 6–12 cm) and for each organ of the plant separately (stem–root, root–leaf, stem–leaf) were interpreted. The interpretation of the data was performed for the control samples as well as for the contaminated samples.

## 3. Results 

### 3.1. Physical and Chemical Properties of Substrate and Watering Media

The results of physical–chemical parameters detected from the soil control showed a conductivity of 475 μS/cm, humus 9.3%, K 1872 mg/kg d.w. (dry weight), total nitrogen (N_total_) 1.20%, total phosphorus (P_total_) 4173 mg/kg d.w., total organic carbon (TOC_total_) 10%, and total carbon (C_total_) 23%. The pH value indicated a weak acid reaction of the soil. Due to nitrogen and phosphorus content, the soil was considered a clay soil that was rich in organic matter. The soil C/N ratio of 19.2 indicated rather mineralization than microbial immobilization, which means microbial activity in soil and the release of nitrogen, available for plant uptake.

No organic chlorinated and triazine pesticides were detected in soil. Co, Cu, Mn, Zn, Cr, V, Mo, and Se were in the limit value for sensitive soil according to the Romanian Law no. 756/1997. However, the following essential elements such as Ca 108,099 mg/kg d.w., Mg 3171 mg/kg d.w., Na 132 mg/kg d.w., and Fe 14,523 mg/kg d.w. were detected.

All the soil samples’ pH values (Mettler Toledo SevenGo) measured before and during the experiments ranged from 6.21 to 6.64 for control and 5.34 to 5.83 for contaminated soil.

The quality of water used for plants watering during the test period corresponded to free chlorine potable water, and no toxic heavy metals were detected. As, Cd, Co, Cr, Ni, Pb, V, Mo, Se, and Sb values were below the method quantification limits. Moreover, Cu 5.1 µg/L, Mn 2.6 µg/L, Fe 39.3 µg/L, Zn 16.3 µg/L, Ca 41.8 µg/L, Mg 4.8 µg/L, and Al 109 µg/L were found in the soaking water.

### 3.2. Metals Concentration in Soil Samples 

**Figure 1:**
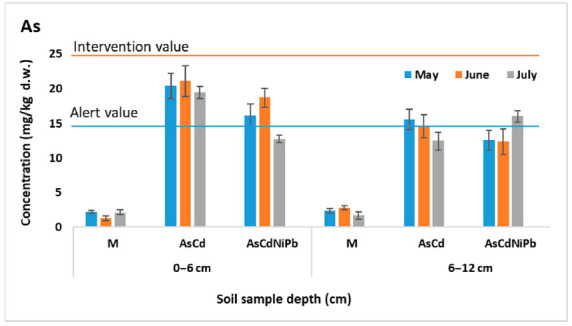
Variation of As concentration in soil (average ± SD, *n* = 6).

**Figure 2:**
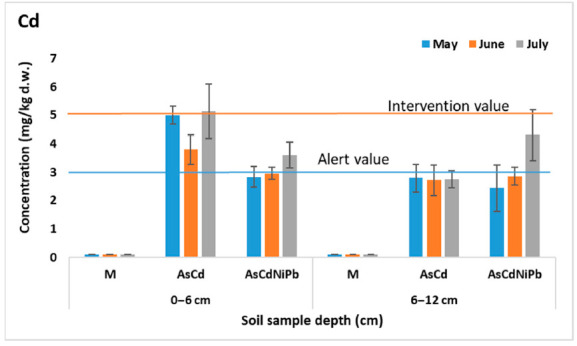
Variation of Cd concentration in soil (average ± SD, *n* = 6).

**Figure 3:**
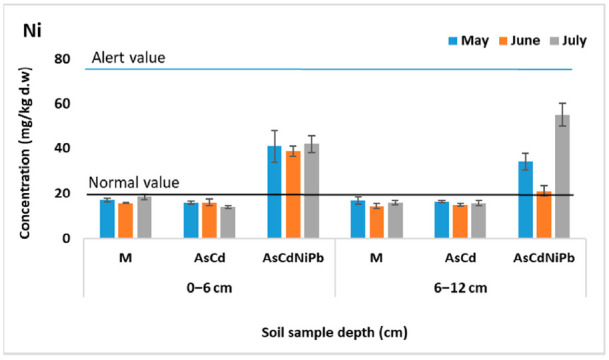
Variation of Ni concentration in soil (average ± SD, *n* = 6).

**Figure 4:**
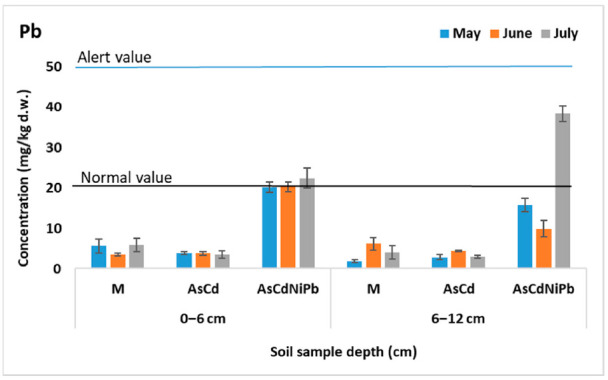
Variation of Pb concentration in soil (average ± SD, *n* = 6).

Figure 1, Figure 2, Figure 3 and Figure 4 present the results of As, Cd, Ni, and Pb concentrations detected in the enriched soil from May to July. After one week of exposure, the contaminated soil samples from the experiments AsCd and AsCdNiPb showed significant metals content compared to the control (*p* < 0.05), which was mainly due to metal soil enrichment. However, metal concentrations (As, Cd, Ni, Pb) were below the intervention threshold for sensitive use, so As and Cd content was above the alert threshold for both AsCd and AsCdNiPb experiments, respectively. In the case of the AsCdNiPb experiment, Ni and Pb concentrations slowly increased over the normal value. The metal concentration detected in the soil samples from May and July in case of the three experiments showed a correlation coefficient of *r* = 0.99, pointing out that the metals accumulation in soil has the same tendency.

Slight differences (*p* > 0.05) were observed between the metals concentration results from the two depths samples (0–6 cm and 6–12 cm).

Generally, at the top soil layer, the metallic elements were found in high concentrations compared to the bottom soil layer for both experiments. Exceptions were observed in case of Ni and Pb with the same distribution between layers in the AsCd experiment similar to those detected in control sample (M). Ni and Pb were added in soil only in experiment AsCdNiPb (in mix with As and Cd). Similarly, these elements increase in the bottom soil layer at the final of test (July) in case of the AsCdNiPb experiment.

The metal concentration differences recorded between the two experiments in May can be explained by: (i) uneven homogenization of the soil with the metal solutions added through watering; (ii) the distribution of metals influenced by the substrate characteristics; and (iii) the plant absorption in a week of exposure.

The remanence of metals in soil at the end of the experiment was as follows: 100% of As, 98% of Cd, 88–95% of Ni, and 92% of Pb. The differences of metals amounts in case of Cd, Ni, and Pb were accumulated by mint plants.

Temporal analyses showed insignificant variations of soil metal concentrations, except for July, when an increase in the bottom soil layer of metals concentrations was observed. The variation was not significant (*p* > 0.05) as a result of metals mobility assisted by soaking.

Moreover, about 20% difference in As concentration between AsCd and AsCdNiPb experiments was noticed, although a similar concentration (23.7 mg/kg d.w.) was added for both experiments. Significant differences in As content at the two depths (*p* = 0.005) in case of AsCd experiment were recorded.

Considering the homogeneity and chemical stability, no significant changes in the metal distribution in control samples between depths and according to the exposure time (*p* > 0.05) were observed.

### 3.3. Metals Concentration in Mint Samples

Control mint plants showed no metal concentration higher than other reported studies or WHO limits. Instead, differences in metal concentration were emphasized. Very significant (*p* < 0.01) differences of Cd and Ni content in the root–stem system and no significant changes (*p* > 0.05) in root–leaves and stem–leaves were recorded. In root–leaves systems, Ni showed significant changes due to the concentration registered in root (*p* = 0.01). In addition, no significant change (*p* > 0.05) was detected between plant organs in controls in case of Pb concentration (Table 3).

The analysis of exposed mint plants showed the presence of toxic metals except for As. The value of As concentration detected in *Mentha piperita* was below the quantification limit of the method in root, stem, and leaves for both experiments (AsCd and AsCdNiPb), although the As content (23.7 mg/kg d.w.) which was added in soil reached the threshold intervention value (25 mg/kg d.w.).

**Figure 5:**
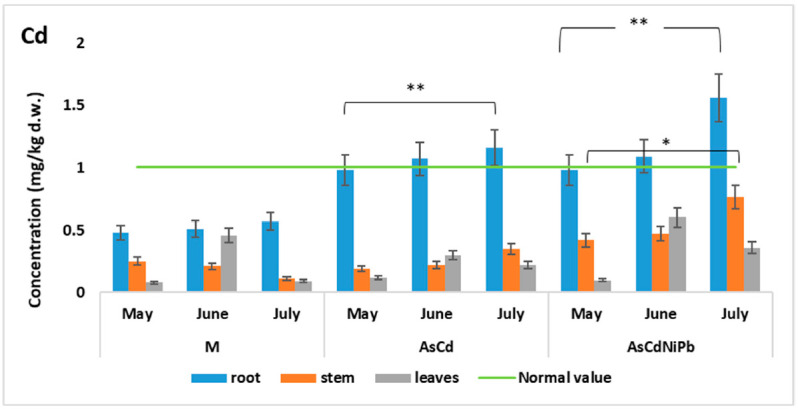
Variation of Cd concentration in *Mentha piperita.* The gray lines on the top indicate significant differences: ** in root (*p* < 0.01) and * in stem (*p* < 0.05), during the exposed period (from May to July) compared to control (M).

Normal values (<1 mg/kg d.w.) of Cd concentrations were found in the aerial parts of the mint plants during the entire experiment period. On the contrary, Cd concentration in root was higher than 1 mg/ kg d.w (AsCd test: 1.07 mg/kg d.w. in June and 1.16 mg/kg d.w in July; AsCdNiPb test: 1.09 mg/ mg/kg d.w. in June and 1.56 mg/kg d.w in July) (Figure 5).

Cd had a higher concentration in stem (in June) and leaves (in July) for AsCdNiPb compared to AsCd in the experiment and exceeded the WHO maximum permissible value (0.03 mg/kg d.w.).

**Figure 6:**
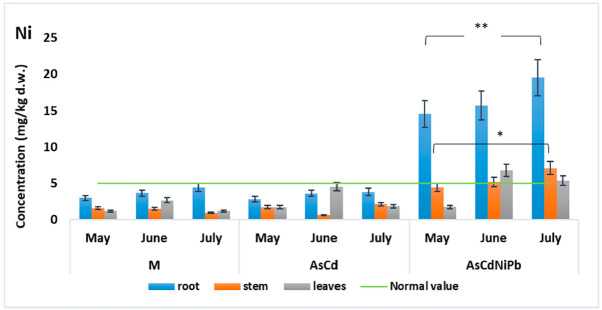
Variation of Ni concentration in *Mentha piperita*. The gray lines on the top indicate significant differences: ** in root (*p* < 0.01) and * in stem (*p* < 0.05), during the exposed period (from May to July) compared to control (M).

**Figure 7:**
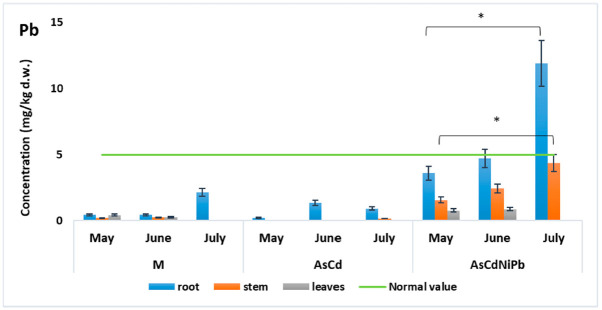
Variation of Pb concentration in *Mentha piperita.* The gray lines on the top indicate significant differences: * in root and stem (*p* < 0.05), during the exposed period (from May to July) compared to control (M).

Slight modifications were observed in case of Ni (Figure 6) and Pb (Figure 7) concentrations in the AsCd experiment compared to the control experiment (M). Additionally, Ni and Pb were added to the soil only in the AsCdNiPb experiment. Both metals Ni (19.52 mg/kg d.w) and Pb (11.88 mg/kg d.w), respectively, had maximum values in the mint root in July, exceeding the limit for the normal plant development (5 mg/kg d.w.). This observation was correlated with the same trend of Ni and Pb values detected in soil in July, where the highest concentrations of this metals were observed. Therefore, the results could be influenced by plant capacity to absorb and retain these metals in different developmental stages and the metals’ bioavailability.

Ni showed a similar behavior as Cd in the root–stem system, accumulating more in roots than in the stems and leaves (*p* < 0.01). Comparing the root–leaves concentrations, Ni showed *p* values > 0.05 in the AsCd experiment and *p* < 0.01 in the AsCdNiPb experiment, both in root, as we expected due to Ni addition (Table 3).

No significant changes were observed for Cd and Ni between stem and leaves.

Pb had a similar behavior as Cd and Ni, accumulating mostly in the root of the plant. Pb concentrations (0.2 to 1.35 mg/kg) in AsCd were similar with the ones detected in the control experiment (M) (0.4 to 2.13 mg/kg). The highest concentration was detected in the root samples in July for the AsCdNiPb experiment (11.88 mg/kg d.w.). The Pb concentrations were significantly different (*p* = 0.03 to 0.04) in roots compared to aerial organs (Table 3).

Comparing the same plant organ in the experiments, we observed very significant (*p* < 0.01) and significant (*p* < 0.05) differences of Cd, Ni, and Pb concentrations between control experiments and contaminated experiments (Table 4) at the root and stem level. There were no changes in Cd and Ni concentrations comparing the data obtained for AsCd and AsCdNiPb. The differences in Ni and Pb were due to the addition of this element in AsCdNiPb. No significant changes were observed for the leaves comparing all the experiment data.

The experiments showed that metals accumulation in mint was as follows: root > stem > leaves.

### 3.4. Biometrical Measurements and Chlorophyll Detection

**Figure 8:**
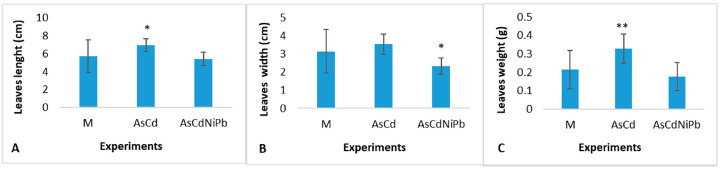
Biometrical data of *Mentha piperita* leaves (after 1 month of exposure), expressed as average ± SD (*n* = 12), where: (**A**)—Leaves length; (**B**)—Leaves width; (**C**)—Leaves weight; and * significant differences (*p* < 0.05), ** very significant differences (*p* < 0.01) compared to control (M)

Figure 8A–C shows the biometrical measurements of mint leaves. The data collected after one month showed significant differences in length (*p* = 0.018)—Figure 8A and weight (*p* = 0.009)—Figure 8C of the leaves foliar surfaces compared to the control in the AsCd experiment. The plants exposure to the AsCdNiPb combination revealed a significant change in leaf surfaces (*p* = 0.021)—Figure 8B correlated with the decrease of weight compared to the control. The stem elongation in control was 30 ± 4 cm, and in tests, it was 27 ± 5 cm.

At the end of the test in June, the mint plants exposed to metals showed phytotoxic effects translated by leaf fall and chlorosis. The stems showed an elongation of 46 ± 7 cm and rare leaves compared to the control plants with a growth of 54 ± 5 cm and an abundance of leaves. It was observed a reduction in the roots size for AsCd and AsCdNiPb tests compared to the control.

**Figure 9:**
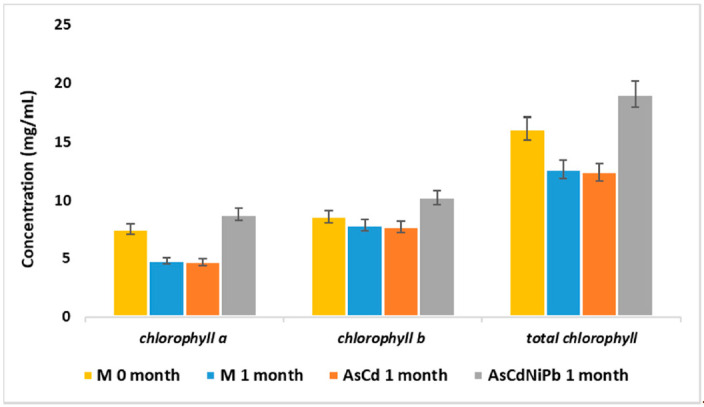
Chlorophyll pigments concentration in *Mentha piperita* leaves, expressed as average ± SD (*n* = 4).

The AsCdNiPb experiment revealed an increase in chlorophyll content (Figure 9) compared to AsCd experiment and control test. Moreover, chlorophyll *b* content was higher compared to chlorophyll *a.* Our results showed that total chlorophyll increased proportionally with chemical elements supplementation.

### 3.5. Transfer Coefficient (TC) and Translocation Factor (TF)

The TC values were calculated as the ratio between the average of metal concentration in the roots divided by the average of metal concentration detected in the soil. The TF values represent the ratio between the average of metal concentration in the aerial part of the plant divided by the average metal concentration detected in the roots.

**Figure 10:**
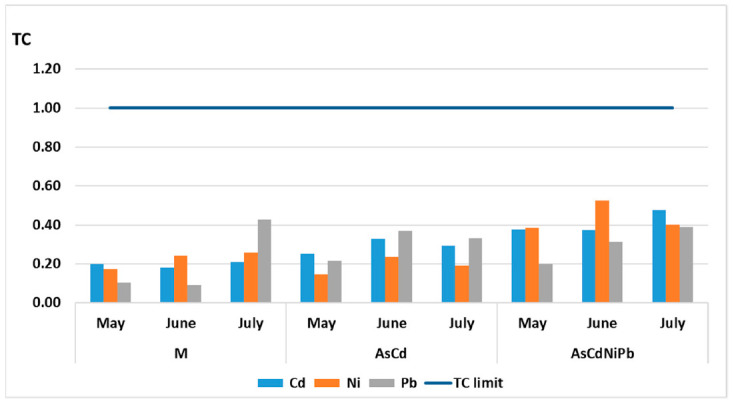
Transfer coefficient (TC) of metals from soil to *Mentha piperita* root.

TC values remained constant for all elements with a slight increase in case of polluted samples (Figure 10). The TC values calculated for Cd, Ni, and Pb were below 1 for both experiments (AsCd and AsCdNiPb). For Cd, the transfer coefficient values in AsCd and AsCdNiPb experiments ranged between 0.2 and 0.48; for Pb, the TC values were between 0.10 and 0.43, and for Ni, the TC values varied between 0.17 and 0.53.

**Figure 11:**
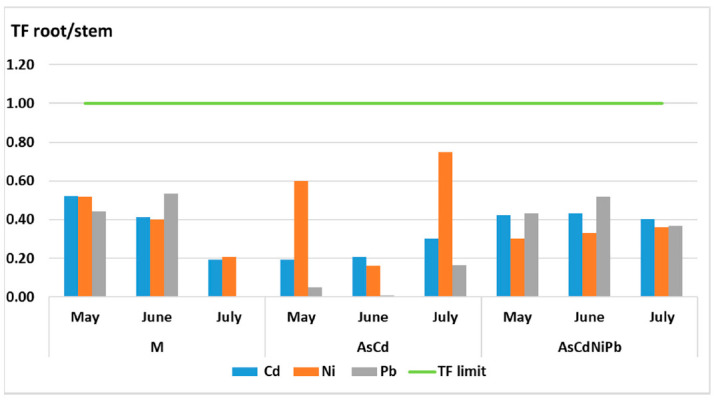
Translocation factor (TF) of metals from root to stem in *Mentha piperita.*

**Figure 12:**
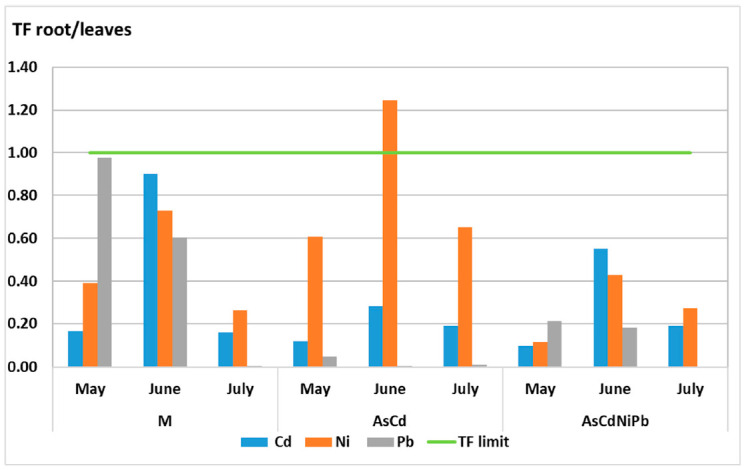
Translocation factor (TF) of metals from root to leaves in *Mentha piperita.*

The studied metals were not efficiently translocated in aerial parts of plants. The TF values were below 1, indicating a non-efficient translocation of metals between organs (Figure 11 and Figure 12). Ni translocation from root to stem increased comparing with Pb in the AsCdNiPb experiment. This indicates the Ni utilization in plant nutrition. Cd translocation in stem was superior in the AsCdNiPb experiment as in case of the AsCd experiment, but it was not significant.

The TF values calculated for root/leaves revealed Ni increased values for all experiments, the maximum TC value being 1.28 in the AsCd experiment. This TC value indicated the transfer of Ni from root to leaves, the average concentrations of 7 mg/kg were situated under a phytotoxic value of 30 mg/kg in plants. Moreover, differences between the pollutants combinations and control (M) were observed. Ni presented lower TF values in the AsCdNiPb experiment (in control range) as to the AsCd experiment. The TF value of Cd increased about 40% in June in the AsCdNiPb experiment in comparison to the same period in the AsCd experiment, but in July, the TF values decreased at 0.19 in both combinations, being in the control range.

## 4. Discussion

### 4.1. Metals Concentration in Mint Samples

Heavy metals uptake by plants is achieved through diffusion or active uptake at the root level by ion exchange between clay and the root surface, which is a process facilitated by different biotic and abiotic factors.

Our results of metals concentration in plant samples were compared with the literature limits. The concentrations of studied metals specified in the literature in plants were As 0.01 to 0.06 mg/kg [8], Cd less than 0.1 to 1 mg/kg, Ni from 0.1 to 5 mg/kg [52], and Pb from 1 to 5 mg/kg [43]. The concentrations that could induce phytotoxic effects in plants are As 2 mg/kg, Cd 10 mg/kg, Ni 30 mg/kg [53], and Pb 20 mg/kg [9]. Moreover, WHO established the maximum permissible limits in food plants as follows: As 1 mg/kg, Cd 0.03 mg/kg, and Pb 10 mg/kg [16].

The plant samples showed different metal concentrations of Cd, Ni, and Pb, except for As. The values of As in *Mentha piperita* grown in laboratory conditions were under detection limits in all plant samples. Some studies reported that the As bioavailable concentration from soil is lower than the total content because As tend to form stable compounds that are less bioavailable and depend on the soil–water–plant system [54]. As is strongly absorbed by Fe, Mn, and Al hydroxides and clays [55]. Furthermore, the use of some inorganic (ferrous sulfate, lamination slags, iron-bearing additives, goethite, metallurgical byproducts, red mud, all based especially on Fe and Mg oxides) or organic biochar obtained from different vegetal, industrial, or municipal wastes) could reduce the mobility of arsenic in plants but induce the decreases of biomass development. Due to low availability in soil, As has been reported in plants in very small concentration of even less 10 μg/kg [56].

The used garden soil and their characteristics regarding the low acidity (pH = 6.7), clay, and the presence of other nutritive components (P_total_ = 4173 mg/kg, N_total_ = 12,055 mg/kg, K= 1872 mg/kg) and essential metals (Cu, Zn, Mg, Ca, Fe, Co, and Ni) could explain the As low availability to plant roots. Some studies report that soil fertilization with phosphorus can induce the accumulation of As in plants [19], but other studies disapprove this observation. The high concentration of phosphorus favors the transfer of phosphates at the roots rather than arsenic compounds due to specific phosphate transporters specifically built for oxyanion, contributing to the reduction of As accumulation in plants [57].

In addition, the used substrate contained an important concentration of Fe (14,523 mg/kg d.w.) and Mn (373 mg/kg d.w.) that could prevent As transfer in plant.

Cd concentration showed changes in the systems root–stem and root–leaves in both experiments. Cd accumulation decreases proportionally with Ni and Pb supplementation in the AsCdNiPb experiment. Similar results were obtained in other studies on *Mentha piperita* where the Cd concentration decreased in plant organs when this element is combined with Pb or Cu, thus indicating a competition between these two metals at the root level [38].

Insignificant changes in Ni and Pb concentrations detected in plants were observed.

It is known that Ni is an essential element involved in the plant growth and development, photosynthesis processes, as well as in the plant nutrition. Therefore, the absorption of Ni by *Mentha piperita* was not influenced by the addition of As and Cd. Plants accumulated Ni mostly in the root, but the concentration detected in the aerial parts (stem and leaves) in the AsCdNiPb experiment exceeded the normal value in plants (5 mg/kg) (Figure 6). The WHO established no maximum permissible limit for Ni in food plants, which was probably due to the benefit of this element for plants development and due to the high phytotoxic limit (30 mg/kg).

Pb concentration did not exceed the maximum value allowed by the WHO (10 mg/kg) and normal value (5 mg/kg) in any parts of the plant that are used for medicinal purposes such as stem and leaves (Figure 7). Similar studies showed that Pb tends to accumulate mostly in plant roots, and only a small amount is transported to the stem and leaves [38,58].

As a general observation, the studied metals were retained mostly in the root rather than the aerial parts. This fact can be explained by the ability of metals as cations (Cd^2+^, Ni^2+^, Pb^2+^) to attach to negative charge sites of root cell walls, thus restricting the transfer from the root to the aerial parts [59].

The experiments (M, AsCd, and AsCdNiPb) pointed out that the rank of metals accumulation in mint samples was as follows: root > stem > leaves. One exception was for Ni and Cd in June, where for all the experiments, the metal accumulation order was root > leaves > stem. The same results were obtained in other studies for *Allium sativum* L. plant [60] and *Mentha spicata* [59].

### 4.2. Biometrical Measurements and Chlorophyll Detection

Growth inhibition is one of the symptoms of metal toxicity. The measurements performed for the mint leaves collected after one month of exposure showed positive changes in biomass and foliar size compared to the control. We assume that the additions of metals could stimulate the plant growth in case of the AsCd experiment and did not have visible effects in case of the AsCdNiPb experiment after one month of exposure. The same observations on plant growth were made in previous studies on *Ocimum basilicum* L. plants grown in a mining-contaminated soil. The presence of metals in different concentrations for a limited exposure time could influence plant development, these being involved in plant metabolic processes. Moreover, the plants could adopt particular survival strategies including a metal detoxification process or could favor the absorption of some metals to the detriment of others, the toxic metals being in competitions with essential metals or other nutrients [27].

The plants’ growth in the AsCdNiPb system showed an increased chlorophyll content after one month of exposure compared to the AsCd experiment, but at the end of the tests, the plants’ state changed. After three months of exposure, phytotoxic effects occurred, namely chlorosis and leaves loss.

Chlorophyll content as an indicator of the photosynthetic system as well as plant robustness can offer responses on the soil quality [23]. Houri et al. stated that the overload of heavy metals could lead to photosynthetic system damage, followed by chlorophyll pigments increase as an adaptation method [61]. After one month of exposure, the enrichment with metals influenced both the photosynthetic process measured by the total chlorophyll concentration (Figure 9) and plant biomass quantified as leaves surface and weight (Figure 8).

Similar phytotoxic effects were reported when Cd prolonged exposure occurs [62,63]. In medicinal plants, metals can compete with the uptake of nutrients N, P, K by the plant, thus leading to macronutrient deficiency followed by chlorosis, growth involution, and leaf necrosis [64].

In addition, Pb (5–50 mg/L) can cause visible symptoms of toxicity causing damage to cell structures, reactive oxygen species (ROS) accumulation, and alteration of essential oils composition [65]. On the contrary, some studies on *Mentha spicata* L. showed no significant changes in oil plant constituents as a specific response to metal contamination [40]. By contrast, it was stated that *Mentha piperita* when exposed to Pb and Cr could improve its yield of essential oil and biomass [66].

### 4.3. Transfer Coefficient (TC) and Translocation Factor (TF)

The metals transfer inside of roots generally occurs through passive diffusion at the cell membrane level or throughout active transfer based on the gradient of concentrations. The active transfer is a natural plant-specific process used for the absorption of essential elements. In addition, it represents the pathway of other toxic available elements. To tolerate the non-essential elements, the plants use different strategies such as morphological, physiological, biochemical, and genetic processes [15].

The metal transfer from soil in mint plants was studied. The data showed that TC values remained constant for all metals being below 1 for both experiments (AsCd and AsCdNiPb). *Mentha piperita* immobilized the metals in root, reducing their toxicity, thus developing a tolerant mechanism. A similar result was obtained for Cd (TF 0.61) in mint subjected to a combination of Cd, Ni, Cr, Pb, and Co. No correlation between TF values of Pb (TF 1.08) and Ni (TF 1.16) was identified, which was probably due to the difference between the substrate characteristics [59].

Since the experiments were performed in high organic matter substrate, it was most likely that Cd, Pb, and As were not well-available for plant roots due to their ability to complex with organic matter and remain soil attached. Some studies reported that *Mentha arvenis* can accumulate Cd and Pb in roots and revealed an antagonistic behavior on the growth and physiological activities in the plant as a result of containing soil organic carbon [23].

The data showed a non-efficient translocation of metals between organs (TF < 1). *Mentha piperita* was classified as an excluder plant for all tested metals (Cd, Ni, Pb) (TC < 1) according to the principles of classification of medicinal plants as hyper-accumulators or excluders [41]. Prasad et al. showed that *Mentha arvenis, Mentha piperita*, and *Mentha citrate* are not hyper-accumulators for Cr and Pb [67]. Our results confirmed that *Mentha piperita* acts as a metal phytostabilizer [67,68,69].

## 5. Conclusions

The present study showed the behavior of one important medicinal plant (*Mentha piperita)* in the presence of several metals (Cd, Ni, Pb, and As) and their capacity to transfer metals to the aerial parts. The laboratory experiments focused on the behavior of mint plants exposed to metals combination, such as AsCd and AsCdNiPb. After three months of exposure, the metals were found in the plants organs as follows: Cd > Ni > Pb > As. In addition, the order of metal accumulation in plant organs was root > stem > leaves.

The plants’ short exposure to metals showed a positive impact on the growth and the leaves’ biometrical parameters. Moreover, as a result of metals supplementation, total chlorophyll concentration increased in the first month of tests as an adaptation mode, which was followed by phytotoxic effects in the final test due to the long time exposure.

The transfer coefficients of metals (Cd, Ni, Pb) from the soil to the plant root were below 1 for both AsCd and AsCdNiPb experiments, respectively. TC values for Cd ranged between 0.2 and 0.48, for Pb 0.10 ÷ 0.43, and for Ni 0.17 ÷ 0.53. *Mentha piperita* immobilized the metals (case of Cd, Ni, Pb) in roots, reducing their toxicity, developing a tolerant mechanism, or excluding the metal absorption by root (in case of Pb, As).

The studied metals were not efficiently translocated in the aerial parts of plants. The translocation factors were below 1, indicating a weak translocation of metals between vegetative organs. Cd and Pb were accumulated mainly in the root. Furthermore, it was observed that Ni was the only element of the four studied metals in which the transfer occurred from the root to the leaves based on their physiological implication. Our results confirmed that *Mentha piperita* acts as a metal phytostabilizer at the root level and is a metal-tolerant plant if it uses a substrate with the described characteristics and the exposure period is no longer than one month. The long time exposure of mint plants to metals induces visible phytotoxic effects as a result of root development reduction.

Care should be given to the migration of metallic elements in parts of the plant used in medicinally human consumption. After the second month of exposure, Cd and Ni were detected in stem above the WHO limit. Medicinal plants grown in soils polluted with metals can raise quality problems of medicinal products, which requires rigorous control before use.

## Figures and Tables

**Table 1:** The results of metal concentrations (average ± SD) detected in control soil.

Metals	Control Soil (M) ± SD (mg/kg d.w.)	Reference Values for Soils with Sensitive Uses (mg/kg d.w.) *		
		Normal Value	Alert Threshold	Intervention Threshold
As	1.29 ± 0.33	5	15	25
Cd	<0.08 **	1	3	5
Ni	13.4 ± 0.12	20	75	150
Pb	4.8 ± 0.56	20	50	100

Note: * MAPPM Order 756/1997; SD—Standard Deviation (*n* = 6); ** value lower than method quantification limit.

**Table 2:** The performance parameters of the applied method.

Metals	LOD, mg/kg	Precision, %	Uncertainty, %	BCR-482		
				Certified Value ± Uncertainty, mg/kg	Determined Value ± Uncertainty, mg/kg	Recovery, %
As	0.25	7.50	15.3	0.85 ± 0.07	0.88 ± 0.13	103.5
Cd	0.02	5.65	12.5	0.56 ± 0.02	0.54 ± 0.07	96.43
Cr	0.02	4.55	13.1	4.12 ± 0.15	4.23 ± 0.55	102.7
Cu	0.02	5.30	12.6	7.03 ± 0.19	6.94 ± 0.87	98.72
Ni	0.04	4.80	12.7	2.47 ± 0.07	2.56 ± 0.33	103.7
Pb	0.50	5.70	14.6	40.9 ± 1.4	39.6 ± 5.8	96.82
Zn	0.03	5.35	12.7	100.6 ± 2.2	96.4 ± 12.2	95.81

**Table 3:** The *p* values applied to plant organs in terms of metals (Cd, Ni, and Pb) in the exposed period (May–July).

Plant Organ System	Experiment	*p* Values		
		Cd	Ni	Pb
Root–stem	M	** (0.002)	** (0.008)	ns (0.135)
	AsCd	** (0.0001)	* (0.014)	* (0.043)
	AsCdNiPb	* (0.022)	** (0.003)	ns (0.110)
Root–leaves	M	ns (0.062)	* (0.018)	ns (0.256)
	AsCd	** (0.0001)	ns (0.255)	* (0.036)
	AsCdNiPb	* (0.010)	** (0.002)	* (0.038)
Stem–leaves	M	ns (0.445)	ns (0.276)	ns (0.280)
	AsCd	ns (0.303)	ns (0.159)	ns (0.186)
	AsCdNiPb	ns (0.169)	ns (0.306)	* (0.030)

Note: ns (insignificant) *p* > 0.05, * significant *p* < 0.05, ** very significant *p* < 0.01.

**Table 4:** The *p* values for the controls and polluted experiments using the data obtained for the same plant organ in the exposed period (May–July).

Plant Organ/Experiment	*p* Values		
	Cd	Ni	Pb
**Root**			
M/AsCd	** (0.001)	ns (0.327)	ns (0.404)
M/AsCdNiPb	** (0.009)	** (0.0005)	* (0.048)
AsCd/AsCdNiPb	ns (0.234)	** (0.005)	ns (0.074)
**Stem**			
M/AsCd	ns (0.191)	ns (0.381)	ns (0.179)
M/AsCdNiPb	* (0.030)	* (0.012)	* (0.040)
AsCd/AsCdNiPb	* (0.049)	** (0.009)	* (0.040)
**Leaves**			
M/AsCd	ns (0.491)	ns (0.197)	ns (0.103)
M/AsCdNiPb	ns (0.247)	ns (0.089)	ns (0.188)
AsCd/AsCdNiPb	ns (0.220)	ns (0.171)	ns (0.095)

Note: ns (insignificant) *p* > 0.05, * significant *p* < 0.05, ** very significant *p* < 0.01.

## Data Availability

The data presented in this study are available on request from the corresponding author. The data are not publicly available due to ongoing research in this field carried out within the above mentioned projects.
